# Nutrient Concentrations Induced Abiotic Stresses to Sweet Pepper Seedlings in Hydroponic Culture

**DOI:** 10.3390/plants11081098

**Published:** 2022-04-18

**Authors:** Xiaotao Ding, Hongmei Zhang, Tingting Qian, Lizhong He, Haijun Jin, Qiang Zhou, Jizhu Yu

**Affiliations:** 1 Shanghai Key Lab of Protected Horticultural Technology, Horticultural Research Institute, Shanghai Academy of Agricultural Sciences, Shanghai 201403, China; xiaotao198108@163.com (X.D.); zhanghongmei@saas.sh.cn (H.Z.); qiantingting@saas.sh.cn (T.Q.); 13851997535@163.com (L.H.); jinhaijun@saas.sh.cn (H.J.); 2 Shanghai Dushi Green Engineering Co., Ltd., Shanghai 201106, China

**Keywords:** photosynthesis, antioxidant enzyme, mineral elements, osmotic stress, root activity

## Abstract

The primary goal of this experiment was to investigate the effects of nutrient electrical conductivity (EC) on the growth and physiological responses of sweet pepper (*Capsicum annuum* L.) in hydroponic culture in a greenhouse. The plant growth parameters, leaf photosynthesis, root activity, soluble protein, malondialdehyde (MDA), proline, activities of antioxidant enzymes (AE), and the contents of plant mineral elements (PME) were measured in six different EC treatments. The results showed that very high or low EC treatments clearly decreased the plant height, stem diameter, shoot dry weight, and leaf net photosynthetic rate, while increasing the content of MDA and the activities of ascorbate peroxidase and guaiacol peroxidase. The contents of proline and soluble protein increased gradually from the low to high EC treatments. The root activities decreased significantly, and the main PME clearly did not increase or even decreased at high EC levels. Very high EC treatments suppressed growth even more than those of very low EC. Treatments that were too low or high EC suppressed plant growth, owing to abiotic stress (either nutrient deficiency or salinity), since the plants had to regulate the activities of AE and increase the accumulation of osmolytes to adjust to the abiotic stresses.

## 1. Introduction

Hydroponics refers to the growth of plants without the use of soil as a rooting medium, and the roots absorb inorganic nutrients from the irrigation water [1]. This type of culture could overcome the problems of barren soil, soil salinity, and soilborne diseases and is more effective at increasing agriculture sustainability, as well as improving environmental health. Hydroponic production has evolved to utilize highly advanced technical systems and methods and has been widely used in protected agriculture to improve the growing environment and provide optimal water and nutrient supply for cultivated crops [2].

One of the biggest challenges in hydroponics is the management of nutrient concentration [3]. The electrical conductivity (EC) is an index of the salt concentrations of nutrients, which is often used to evaluate the nutrient status in hydroponic solutions in greenhouse plant production [4]. EC is also an indicator of the osmotic pressure of the solution, which is related to the total composition of nutrient ions available to plants in the root zone [5]. Many growers use EC to adjust the salinity of substrates to improve the quality of the fruit, which may reduce vegetative development, as well as the water-use efficiency and yield [6]. Using an optimal EC is critical for different types of plant growth and yields [7]. A higher EC beyond the threshold levels often hinders nutrient uptake and reduces crop production by increasing osmotic pressure, although a lower EC may severely decrease plant health and yield [8,9].

Sweet pepper (*Capsicum annuum* L.), which originated from Central and South America, is an important vegetable crop and thermophilic species in the world. Sweet pepper was introduced to Europe during the 16th century from Spanish and Portuguese expeditions and then gradually spread all around the world [10]. There are many studies that focus on factors that affect sweet pepper growth, the level of fertilization and the selection of varieties [11], yield and fruit quality [12,13], disease [14], and abiotic and biotic stresses [15]. Some researchers have also conducted research on the management of nutrient solutions (NS) to grow sweet peppers [16,17,18], but the experimental design for the range of electrical conductivity (EC) values was small. Thus, these conditions may not accurately reflect the potential for plant tolerance and adequately reveal the effects of different levels of EC on sweet pepper growth and its physiological responses. Moreover, limited research has focused on the definite changes of mineral elements under different levels of EC.

The purpose of this study was to investigate the effects of different EC of nutrients on plant growth, leaf photosynthesis, root activity, antioxidant enzyme (AE) activities, and the changes of plant mineral element (PME) content in sweet pepper seedlings. Moreover, we attempted to find the optimal EC and underlying mechanisms that sweet pepper seedlings adjusted in response to abiotic stresses in too low or high EC levels.

## 2. Results

### 2.1. Plant Growth Analysis

**Figure 1:**
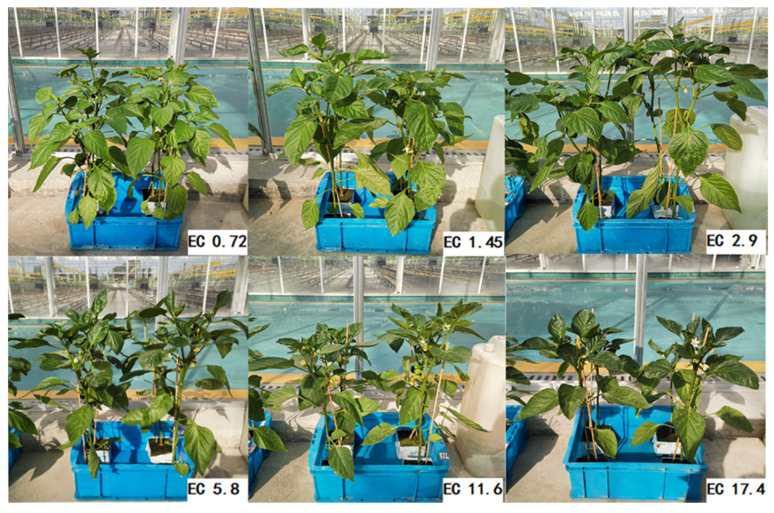
Pictures of plants in the pots of different EC treatments after 25 days of treatment. EC0.72, EC1.45, EC2.9, EC5.8, EC11.6, and EC17.4 represent the different nutrient solution electrical conductivities of 0.72 dS m^−1^, 1.45 dS m^−1^, 2.9 dS m^−1^, 5.8 dS m^−1^, 11.6 dS m^−1^, and 17.4 dS m^−1^.

Among all the EC treatments, the plant height and stem diameter of the EC1.45 and EC2.9 treatments were significantly higher than those of the other treatments, followed by EC0.72 and EC5.8, then EC11.6, and the lowest value was that of the EC17.4 treatment (Table 1; Figure 1). The EC2.9 treatment contained the highest values of shoot fresh weight (FW), dry weight (DW), and shoot moisture content (MC), and the values gradually decreased from EC1.45 to EC5.8 and EC0.72, EC11.6, and EC17.4. Compared with the EC2.9 treatment, the plant height, stem diameter, FW, DW, and MC were reduced by 37.2%, 21.5%, 59.5%, 43.0%, and 6.0%, respectively, in the EC17.4 treatment, by 27.9%, 12.3%, 48.9%, 34.4%, and 4.6%, respectively, in the EC11.6 treatment, and by 8.7%, 6.2%, 24.6%, 16.5%, and 1.6%, respectively, in the EC0.72 treatment (Table 1).

### 2.2. Leaf Gas Exchange Analysis

**Figure 2:**
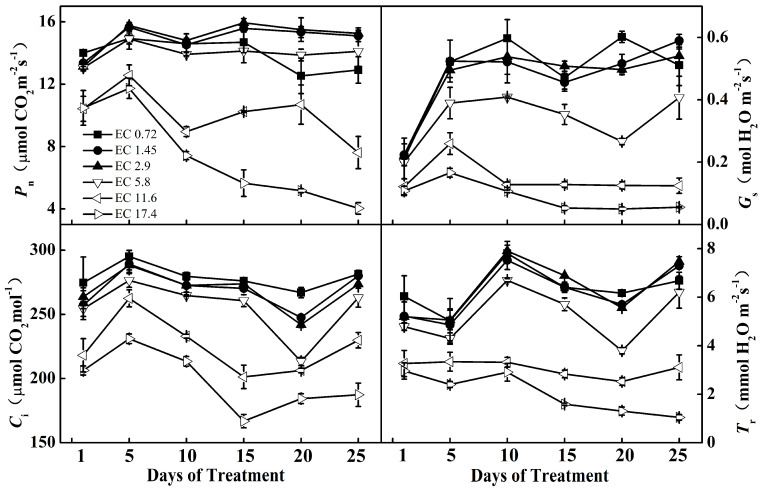
Effects of different EC treatments on the net photosynthetic rate (*P*_n_), stomatal conductance (*G*_s_), intercellular CO_2_ concentration (*C*_i_), and transpiration rate (*T*_r_) after 1 d, 5 d, 10 d, 15 d, 20 d, and 25 d of treatment. Data are the means of at least three biological replications. EC0.72, EC1.45, EC2.9, EC5.8, EC11.6, and EC17.4 represent the different nutrient solution electrical conductivities of 0.72 dS m^−1^, 1.45 dS m^−1^, 2.9 dS m^−1^, 5.8 dS m^−1^, 11.6 dS m^−1^, and 17.4 dS m^−1^.

The net photosynthetic rate (*P*_n_), stomatal conductance (*G*_s_), intercellular CO_2_ concentration (*C*_i_), and transpiration rate (*T*_r_) were significantly influenced by the different EC treatments (Figure 2). The repeated measure ANOVA was used to test the significant effects of the EC treatments, days of measurement, and their interactions on these parameters. The ANOVA test results are shown in Appendix A Table A1, in which all of the *p* values for EC treatment were <0.001. In comparison with the other treatments, the *P*_n_, *G*_s_, *C*_i_, and *T*_r_ of EC11.6 and EC17.4 had decreased dramatically one day after the treatment. These parameters remained at low levels during the following treatment days. Five days after the treatment, there were clear differences in the leaf gas exchange parameters between the EC11.6 and EC17.4 treatments, particularly for *P*_n_ (*t* value = 5.39, *p* < 0.0001) and *C_i_* (*t* value = 6.33, *p* < 0.0001). The decrease of *P*_n_ in the plants treated with EC17.4 was even more apparent, which decreased by 66% compared with the *P*_n_ in 5 d. The growth of plants in the EC11.6 and EC17.4 treatments was remarkedly inhibited, as the top leaves became small; the leaves were dark, and there was a phenomenon of “blunt with blossom” (Figure 1), which may be related to the obvious reduction of its photosynthetic gas exchange parameters. The *P*_n_ of the EC5.8 treatment was in a relatively normal range during the treatment period, but the color of the new leaves darkened, and *G*_s_, *C*_i_, and *T*_r_ were also clearly reduced. The plants of the EC2.9 and EC1.45 treatments were in good condition for photosynthesis with the *P*_n_, *G*_s,_ *C*_i_, and *T*_r_ all at high levels. The *P*_n_ of the EC0.72 plants was relatively normal in the early stage but decreased slightly by 15% at 20 days compared with 15 days of the treatment.

### 2.3. Analyses of the Contents of Leaf Chlorophyll (Chl) and Carotenoids (Car)

**Figure 3:**
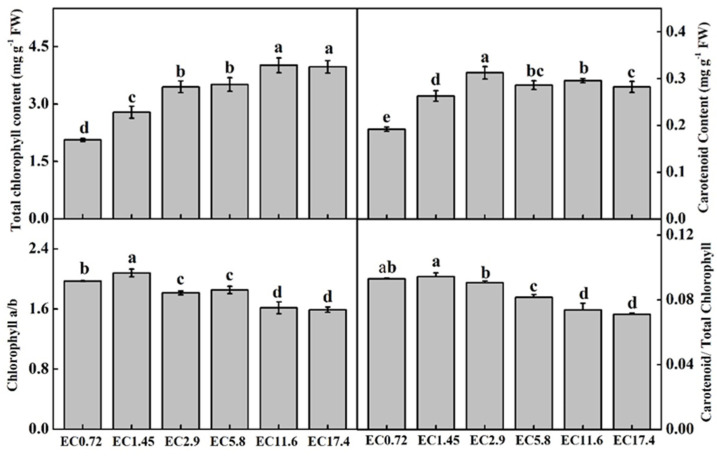
Effects of different EC treatments on the total chlorophyll content, carotenoid content, chlorophyll a/b, and carotenoid/total chlorophyll of different treatments after 25 days. EC0.72, EC1.45, EC2.9, EC5.8, EC11.6, and EC17.4 represent the different nutrient solution electrical conductivities of 0.72 dS m^−1^, 1.45 dS m^−1^, 2.9 dS m^−1^, 5.8 dS m^−1^, 11.6 dS m^−1^, and 17.4 dS m^−1^. Data represent the mean ± SD (*n* = 3). Different letters indicate significant differences at *a* = 0.05 based on the Least Significant Difference test.

The total contents of Chl increased in parallel with the EC, while the highest value appeared in the EC11.6 treatment, and there was no significant difference between the EC11.6 and EC17.4 treatments (Figure 3). The contents of Car increased from the EC0 to EC2.9 treatments and then decreased in the EC5.8, EC11.6, and EC17.4 treatments. The lowest value was in the EC0.72 treatment. There were similar changes in the ratios of Chl a/b and Car/total Chl for the different EC treatments. The highest ratios of Chl a/b and Car/total Chl were in the EC1.45 treatment followed by the EC0.72, EC2.9, and EC5.8 treatments, and the lowest were in the EC11.6 and EC17.4 treatments (Figure 3).

### 2.4. Root Activity Analysis

**Figure 4:**
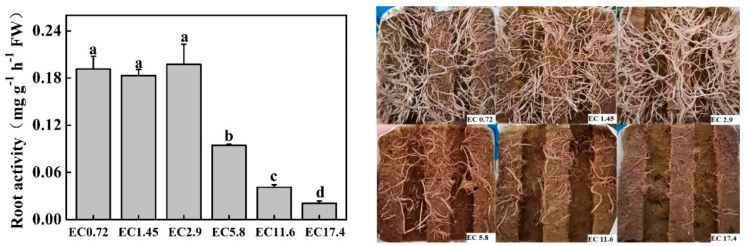
Effects of different EC treatments on root activity, and pictures of their roots after 25 days of treatment. EC0.72, EC1.45, EC2.9, EC5.8, EC11.6, and EC17.4 represent the different nutrient solution electrical conductivities of 0.72 dS m^−1^, 1.45 dS m^−1^, 2.9 dS m^−1^, 5.8 dS m^−1^, 11.6 dS m^−1^, and 17.4 dS m^−1^. Data represent the mean ± SD (*n* = 3). Different letters indicate significant differences at α = 0.05 based on the Least Significant Difference test.

Root respiration was used as an indicator of root activity. The stronger the respiration, the stronger the root activity. The highest root activity was found in the EC2.9 treatment, and there were no significant differences among the EC0.72, EC1.45, and EC2.9 treatments (Figure 4). The root activities of the EC5.8, EC11.6, and EC17.4 treatments deceased significantly by 52.0%, 79.1%, and 89.4%, respectively, compared with the EC2.9 treatment. The root pictures effectively demonstrated the root activity of different treatments (Figure 4), which also reflected the growth of their shoots (Figure 1).

### 2.5. AE Analysis

**Figure 5:**
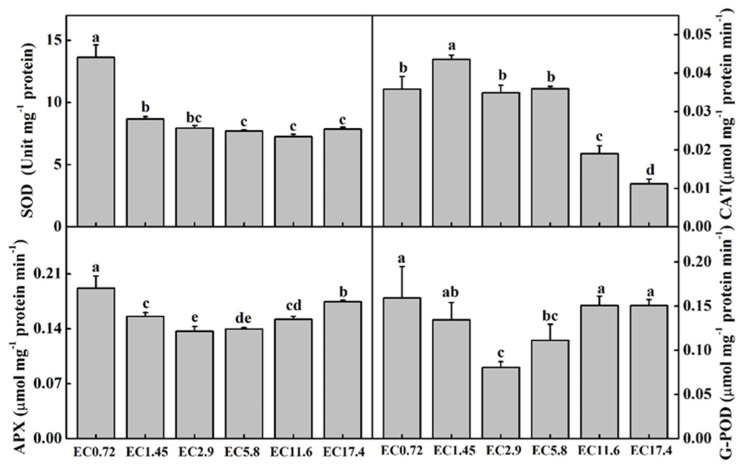
Effects of different EC treatments on the antioxidant enzyme activities of SOD, CAT, APX, and G-POD after 25 days of treatment. Data represent the mean ± SD (*n* = 3). EC0.72, EC1.45, EC2.9, EC5.8, EC11.6, and EC17.4 represent the different nutrient solution electrical conductivities of 0.72 dS m^−1^, 1.45 dS m^−1^, 2.9 dS m^−1^, 5.8 dS m^−1^, 11.6 dS m^−1^, and 17.4 dS m^−1^. Data represent the mean ± SD (*n* = 3). Different letters indicate significant differences at α = 0.05 based on the Least Significant Difference test. APX = ascorbate peroxidase; CAT = catalase; G-POD = guaiacol peroxidase; SOD = superoxide dismutase.

The enzymatic activities of superoxide dismutase (SOD), catalase (CAT), ascorbate peroxidase (APX), and guaiacol peroxidase (G-POD) differed significantly among the EC treatments (Figure 5). The activities of SOD were the highest in the EC0.72 treatment. Those of the EC1.45 treatment were significantly lower than those of the EC0.72 treatment, and the activities of SOD in all the other treatments were lower. The activities of CAT were the highest in EC1.45 among all the treatments. The EC0.72, EC2.9, and EC5.8 treatments exhibited no significant difference, and the EC11.6 and EC17.4 treatments decreased the activities of CAT, with the lowest values in the EC17.4 treatment. The activities of APX and G-POD changed similarly among all the treatments, and the lowest values were in the EC2.9 treatment. Their activities gradually increased in the high and low EC treatments, and there was no significant difference between the EC2.9 and EC5.8 treatments.

### 2.6. Analyses of the Contents of Soluble Protein, MDA, and Proline

The soluble protein content of the EC11.6 treatment had the highest value among all the treatments, followed by the EC17.2 and EC5.8 treatments, then by the EC2.9 and EC1.45 treatments, with the lowest value identified in the EC0.72 treatment (Table 2). The MDA content of the EC17.4 treatment was the highest, and the EC2.9 treatment had the lowest content of MDA. A high EC resulted in a more apparent increase in MDA than a low EC. The contents of proline gradually increased from low to high EC. The EC17.4 treatment had a significantly higher value than those of the other treatments. When the EC value was higher than 2.9 dS m^−1^, the content of proline increased dramatically by 2.4-, 11.3-, and 15.6-fold when the EC5.8, EC11.6, and EC17.4 treatments were compared with the EC2.9 treatment, respectively.

### 2.7. PME Content Analysis

**Figure 6:**
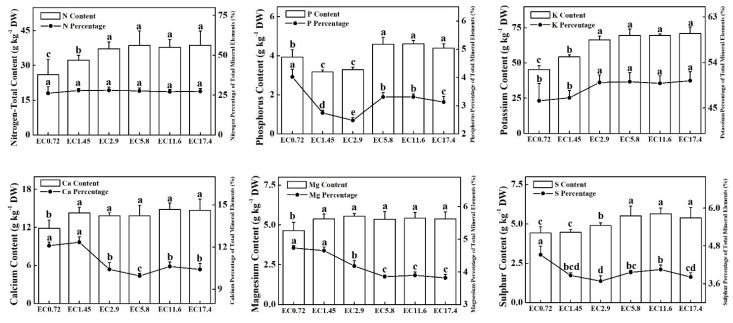
Effects of different EC treatments on the contents of macro plant mineral elements (PME) and their percentage of total PME after 25 days of treatment. EC0.72, EC1.45, EC2.9, EC5.8, EC11.6, and EC17.4 represent the different nutrient solution electrical conductivities of 0.72 dS m^−1^, 1.45 dS m^−1^, 2.9 dS m^−1^, 5.8 dS m^−1^, 11.6 dS m^−1^, and 17.4 dS m^−1^. Data represent the mean ± SD (*n* = 3). Different letters indicate significant differences at α = 0.05 based on the Least Significant Difference test.

**Figure 7:**
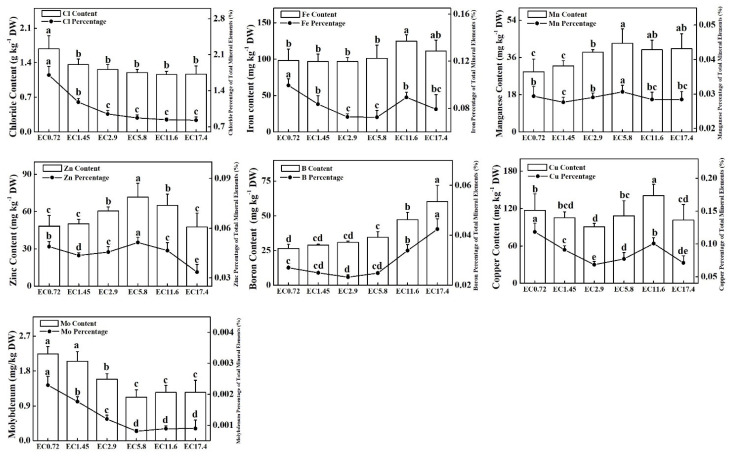
Effects of different EC treatments on the contents of micro plant mineral elements (PME) and their percentage of total PME after 25 days of treatment. EC0.72, EC1.45, EC2.9, EC5.8, EC11.6, and EC17.4 represent the different nutrient solution electrical conductivities of 0.72 dS m^−1^, 1.45 dS m^−1^, 2.9 dS m^−1^, 5.8 dS m^−1^, 11.6 dS m^−1^, and 17.4 dS m^−1^. Data represent the mean ± SD (*n* = 3). Different letters indicate significant differences at α = 0.05 based on the Least Significant Difference test.

The EC treatment had significant effects on the PME content in sweet pepper (Figure 6 and Figure 7). The contents of total nitrogen (TN) and potassium (K) increased as the EC increased, and the EC2.9, EC5.8, EC11.6, and EC17.4 treatments did not differ significantly. The contents of phosphorus (P) in the EC5.8, EC11.6, and EC17.4 treatments were significantly higher than those of the EC0.72 treatment, and that of the EC0.72 treatment was significantly higher than those of the EC1.45 and EC2.9 treatments. The contents of calcium (Ca) and magnesium (Mg) changed similarly among the EC treatments with the lowest value of the EC0.72. There were no significant differences among the other EC treatments. High contents of sulfur (S) and manganese (Mn) appeared in the EC5.8, EC11.6, and EC17.4 treatments and gradually decreased in parallel with the EC. The EC11.6 treatment produced the highest contents of iron (Fe) and copper (Cu). There was no significant difference in the content of Fe among the other EC treatments, and the EC2.9 treatment had the lowest content of Cu. The highest content of zinc (Zn) was in the EC5.8 treatment, followed by those in EC2.9 and EC11.6. The lowest values were in the EC0.72, EC1.45, and EC17.4 treatments. The highest contents of chloride (Cl) and molybdenum (Mo) were in the EC0.72 treatment, and there was no significant difference in the amount of Cl in the other treatments, while the contents of Mo gradually decreased as the EC decreased. The content of boron (B) increased as the EC increased, and the EC17.4 treatment had the highest value. The highest percentage of microelement was Cl, and the lowest was Mo for all the treatments. In most cases, the percentage of microelements in the total PME changed similarly as the content of microelements changed in the different EC treatments (Figure 7). The percentages of P and S of the total PME were the significantly highest in the EC0.72 treatment, and the comparable percentages of Ca and Mg were the significantly highest in the EC0.72 and EC1.45 treatments (Figure 6).

## 3. Discussion

Plant growth has been shown to be affected by the nutrient concentrations in hydroponic culture systems [19,20]. This study found that the plant height, stem diameter, FW, DW, and MC of sweet pepper gradually increased with the increase in EC and had the highest values in the EC2.9 treatment. The high EC treatments of EC11.6 and EC17.4 resulted in lower plant height, stem diameter, FW, DW, and MC, which could be owing to toxicity in the very high salinity NS [8]. The very high EC treatments suppressed the growth of sweet pepper more than the very low EC treatments. A similar result was obtained by Lam et al. [20], who treated *Agastache rugosa* with six EC treatments of 0.5, 1.0, 2.0, 4.0, 6.0, and 8.0 dS m^−1^ and found that almost all the plant growth parameters, such as leaf length, leaf width, leaf area, and stem length, were maximized at 2.0 and 4.0 dS m^−1^ and minimized at 8.0 dS m^−1^ compared with the other EC treatments. Similarly, Albornoz and Lieth [21] found that a high concentration of nutrients (EC of 6 and 10 dS m^−1^) in the root zone significantly decreased the biomass of lettuce (*Lactuca sativa*) because the high concentration of salinity reduced the osmotic potential in the NS. A high EC may reduce the water uptake, turgor pressure, and the retention of toxic ions in the root zone, which results in limited cell expansion and an ion imbalance [20] and finally limits the growth of sweet pepper. In this experiment, we found that the plants became blunt with blossoms in the high EC treatments (EC of 11.6 and 17.4 dS m^−1^). Particularly for the treatment of EC17.4, the plant almost stopped its growth as the treatment days were prolonged, which resulted in no fruits harvested.

Photosynthesis provides energy and carbon assimilation for plant growth and reproduction, which is the fundamental physiological process of plants. A reduction in leaf photosynthesis often leads to a low production of assimilates [22]. In this study, we found that *P*_n_, *G*_s,_ *C*_i_, and *T*_r_ of the EC11.6 and EC17.4 treatments had dramatically decreased one day after treatment with different EC, and these parameters remained at low levels on the following treatment days. This was particularly true for the EC17.4 treatment. *P*_n_ and *G*_s_ gradually decreased to 4 µmol CO_2_·m^−2^s^−1^ and 0.055 mmol H_2_O·m^−2^s^−1^, respectively, 25 days after treatment. This indicated that the sweet pepper had suffered serious salinity stress in the high EC treatment, which could be owing to a decrease in stomatal closure [23,24]. After 15 days of the EC treatments, the *P*_n_ of the EC0.72 and EC5.8 treatments clearly decreased, and the decreases were sustained during the following treatment days. The *P*_n_ of EC5.8 decreased in parallel with those of *G*_s,_ *C*_i_, and *T*_r_ during the 15–25 days of treatments. However, *G*_s,_ *C*_i_, and *T*_r_ of the EC0.72 treatment remained at high levels during the final treatment days, which indicated that other non-stomatal limitations could be a main cause of a reduction in photosynthesis [25]. The plants of the EC2.9 and EC1.45 treatments had higher *P*_n_, *G*_s,_ *C*_i_, and *T*_r_, which indicated that the plants grew well in such EC NS. The photosynthetic parameters of different treatments could be good indicators of their different growth biomass.

It is well known that Chl and Car play important roles in light harvesting, stabilization of the thylakoid membranes, and energy transduction [26,27]. In this study, the total contents of Chl increased with the EC up to EC11.6 and EC17.4. In addition, the contents of Car increased as the EC increased to EC2.9. There were slight decreases in the EC5.8, EC11.6, and EC17.4 treatments, which indicated that sweet pepper can suffer salinity stress at high EC treatments [25,28]. An EC that was too low decreased the contents of Chl and Car, which could be owing to the inadequacy of PME, such as N, Mg, and Fe, which are important factors for the synthesis of Chl and Car and the maintenance of chloroplast structure and function [20,29]. Moreover, an EC that was too high led to decreases in Chl a/b and Car/total Chl, which could indicate that the plants had suffered stress damage during the treatment period [25].

Soluble proteins play an important role in the growth of the plants and are highly important osmotic regulators [30]. In this study, the increase in contents of soluble proteins during the high EC treatment was probably owing to the effects of salinity stress on the plants, which would subsequently produce more soluble proteins as osmotic regulators to detoxify the reactive oxygen species (ROS) synthesized during stress responses [31]. The extremely low treatment of EC0.72 could not supply enough PME to support the production of soluble proteins.

MDA is a widely used marker of oxidative lipid injury whose concentration varies in response to biotic and abiotic stresses [32]. The content of MDA significantly increased in the high EC treatments, such as EC11.6 and EC17.4, which indicates that the plants suffered from a high level of lipid peroxidation, since MDA appears to be the most mutagenic product of lipid peroxidation [33]. The content of MDA was always found to be higher in plants that are sensitive to salinity compared with those that are tolerant to salinity [34]. Talhouni et al. [35] studied eggplant (*Solanum melongena*) at salinity levels of EC of 6–7 dS m^−1^ and found that the content of MDA increased compared with the control of an EC of 1.8–2 dS m^−1^ treatment. Grafting the eggplants alleviated the negative effects of salinity by enhancing their enzymatic antioxidant defense system and resulting in the more efficient uptake of nutrients.

In this study, the content of proline dramatically increased when the EC value exceeded 2.9 dS m^−1^, particularly for the EC11.6 and EC17.4 treatments. The contents of proline in the EC11.6 and EC17.4 treatments increased 11.3- and 15.6-fold compared with the EC2.9 treatment, respectively, which indicates that the sweet pepper in the EC11.6 and EC17.4 treatments suffered serious salinity stress. A similar result was found by Ahmadi and Souri [36], who studied two treatments of EC of 5 and 8 dS m^−1^ of Hoagland NS using various salt combinations that showed that the content of proline in the EC8 treatment was always higher than that of the EC5. Arough et al. [31] also found that the proline content increased in parallel with the EC in different fertilizer treatments. This could be owing to the overproduction of proline, which, in turn, imparted stress tolerance by modulating the ROS to be within normal ranges, which prevents electrolyte leakage by stabilizing membranes, and maintaining cell turgor or osmotic balance, thus preventing an oxidative burst in the plants [37,38].

SOD, CAT, APX, and G-POD are important AE that reduce the ROS to relieve injury to the plant [39]. In this study, the activities of APX and G-POD were high in the low and high EC treatments, indicating that these enzymes had significant roles in minimizing the stress effect by scavenging the ROS [40]. As one of the AsA-glutathione cycle enzymes, APX possesses a high affinity for hydrogen peroxide (H_2_O_2_) and can exert its functions even at low levels of ROS [41]. G-PODs, located in the cytosol, vacuole, cell wall, and apoplast, are involved in important metabolic reactions, such as controlling cell growth, inducing defense mechanisms, and taking part in a range of processes related to stress induced by ROS [39,42]. The activity of SOD in the EC0.72 treatment was higher than that in the other treatments, which could be owing to the frontline protection provided by SOD against ROS by its ability to convert superoxide anions (O_2_^−^) to H_2_O_2_ in the antioxidant defense system [43]. The activities of CAT decreased in the EC11.6 and EC17.4 treatments, and low and middle EC treatments exhibited some high activities, probably because the activity of CAT is only efficient when high levels of H_2_O_2_ are present. This is because its affinity for H_2_O_2_ is relatively lower than that of other AE, such as APX and other types of peroxidases [41].

Plant development is directly correlated with root activity such as the capacity to absorb and transport water and nutrients. We used root respiration as an indicator, as it reveals the metabolic capability of the roots and directly affects plant growth and stress resistance [44]. In this study, the root activity of sweet pepper decreased significantly when the EC ≥ 5.8 dS m^−1^, which indicated that the actual uptake of nutrients or water by roots was obstructed [45]. Such an improper condition might limit the growth of plants by decreasing the activity of roots and reducing their metabolism and nutrient uptake [46]. This could also be the main reason that growth and photosynthesis decreased in the EC17.4, EC11.6, and EC5.8 treatments, since root activity is often positively correlated with the photosynthetic rate and plant growth [47,48]. A similar result was observed by Xuan et al. [49] that the root lengths of both types of turnip rape (*Brassica rapa*) seedlings decreased significantly as the concentration of salt stress increased. Moreover, salt stress decreased the root biomass and increased the MDA content and AE activities of the roots.

Higher contents of N, P, K, Ca, Mg, S, Fe, Mn, Zn, and Cu were observed in sweet pepper plants during the high EC treatment. However, when the EC reached ≥5.8 dS m^−1^, the PME of the plants did not increase or even decreased. Ahmadi and Souri [36] found a similar result that high EC may not increase the macronutrient and micronutrient concentrations of chili pepper plants but increased the concentration of proline in the leaves and the activities of CAT and POD. EC treatments that are too high may result in salinity stress for plants [50], and it is well known that salinity changes the nutrient composition and ratios in plant tissues, resulting in nutrient imbalances [51,52], and can significantly reduce the concentrations of nutrients, such as Ca, K, Mg, and Zn, in the leaves [53]. The salinity stress of high EC also resulted in low root activity of the plant. In addition, nutrients have antagonistic interactions when some are applied at higher levels [54]. This study also found that low EC treatments increase some of the percentage of macroelements of total PME, particularly those of Ca and Mg. Similar results were found by Stanghellini et al. [55] that the accumulation of Ca increased in a single sweet pepper plant treated with a low EC, but the efficiency of water uptake clearly decreased. From the analysis of the contents of PME and percentage of elements of total PME, the precise control of EC was important for the effective absorption of elements and uptake of water.

The growth parameters, Chl, Car, and photosynthesis, directly reflected the effects of the different EC treatments. Root activity, MDA, soluble proteins, and proline changes provided an in-depth explanation of the plant response and adjustment to high EC stress, and antioxidant enzymes activities comprised the defense system that could be activated when EC treatments were too low and too high. The PME explained that too much fertilizer would not be effectively absorbed and leads to salinity stress, while the inadequacy may result in the suppression of plant morphogenesis.

## 4. Materials and Methods

### 4.1. Plant Materials and Experimental Design

The experiment was conducted in a semi-closed Venlo-type glass greenhouse at the Chongming base of the National Engineering Research Center of Protected Agriculture (31°34′ N, 121°41′ E), Shanghai Academy of Agriculture Sciences (Shanghai, China) in 2019–2020. The sweet pepper (*Capsicum annuum* L.) variety used in this study was Stayer RZ (Rijk Zwaan Company, De Lier, Netherlands). Sweet pepper seeds were sown in Grodan blocks (10 cm × 10 cm × 6.5 cm) in a well-heated greenhouse. The sweet peppers grew under natural light, and the growth temperature was established at approximately 25 °C (the greenhouse was cooled when the air temperature was >25 °C) during the day and 17 °C at night (heating began when the greenhouse temperature was <17 °C). The relative humidity of the greenhouse was approximately 50–90%. We chose four plants in which the 10th true leaf had fully expanded as a unit and transplanted them into a plastic container (27 cm × 40 cm × 12 cm) for different EC treatments of nutrient solution (NS). The six EC treatments in this study were arranged, and the experiment was repeated five times. The base mother NS (A and B) was referenced to Hoagland’s solution and was revised following the advice of Eurofins (branch company of Suzhou, China), with the nutrient concentrations listed in Table 3. The NS for the EC treatments were diluted from the mother NS A and B. The plants were grown under the different EC treatments for 25 days, and the largest new leaves of each treatment were harvested at the end of the experiment. We immediately froze the leaf samples in liquid nitrogen and stored them at −80 °C for further analysis.

### 4.2. NS Treatments

The different EC treatments were measured using a portable conductivity meter (DDB-303A; Shanghai Leici, Shanghai, China), and the pH value was determined using pH meter (PHB-4; Shanghai Leici, Shanghai, China). The experiment fertilizers originated from Shanghai Wintong Chemicals Co., Ltd. (Shanghai, China). The six different EC treatments were designed as described by Ding et al. [8], and the detailed settings are shown below: (1) diluted mother NS A and B with deionized water to 0.72 dS m^−1^ of EC (EC0.72), (2) to 1.45 dS m^−1^ of EC (EC1.45), (3) to 2.9 dS m^−1^ of EC (EC2.9), (4) to 5.8 dS m^−1^ of EC (EC5.8), (5) to 11.6 dS m^−1^ of EC (EC11.6), (6) to 17.4 dS m^−1^ of EC (EC17.4). The same amounts of mother nutrient solutions A and B were used for every EC treatment. Hydrochloric acid (HCl) and sodium hydroxide (NaOH) were used to adjust the pH of NS to 5.5 for all the treatments. All the plants were irrigated with an EC of 2.5 dS m^−1^ and a pH of 5.5 before the different EC treatments started. The sweet pepper plants were irrigated approximately three times a week to maintain stable EC levels for all the treatments during the experiment. We irrigated 3 L of different EC nutrient solutions to the growth containers each time and drained the remaining solution after an hour.

### 4.3. Measurements of Sweet Pepper Plant Height, Stem Diameter, Shoot Fresh Weight (FW), Dry Weight (DW), and Shoot Moisture Content (MC)

The sweet pepper plant height, stem diameter, and FW were measured after 25 days of the different EC treatments. The shoots were heated in an oven at 105 °C for 2 h to inactivate the enzymes and then dried at 80 °C over three days to measure the DW. The MC = (1−DW/FW) × 100%. We measured at least three replicates per pot in each treatment.

### 4.4. Measurements of Leaf Gas Exchange Parameters

A Li-6400 Portable Photosynthesis System (LI-COR, Inc., Lincoln, NE, USA) was used to measure the leaf gas exchange parameters of the net photosynthetic rate (*P*_n_), stomatal conductance (*G*_s_), intercellular CO_2_ concentration (*C*_i_), and transpiration rate (*T*_r_). Light was set at 800 µmol photons m^−2^s^−1^, and CO_2_ concentration was set at 400 µmol mol^−1^ for measurements. The air temperature and relative humidity used were those of the greenhouse conditions. Fully developed leaves on the upper and middle of different treatments were randomly selected each time and acclimated to the irradiance level for approximately 2 min before recording. The measurements were conducted at approximately 10 AM at 1, 5, 10, 15, 20, and 25 days of treatment.

### 4.5. Measurements of Leaf Chlorophyll Content, Carotenoid Content, and Root Activity

The supernatant of 0.1 g of leaf tissues was soaked in and extracted with 95% *v*/*v* ethanol (10 mL), until the leaf tissues became completely white, and then measured with a UV-visible spectrophotometer (Ultraviolet-2700; Shimadzu, Tokyo, Japan) at 665, 649, and 470 nm. The contents of chlorophyll (Chl) a, b and carotenoids (Car) were calculated as described by Jiang et al. [25], with modifications.

The root activity was determined using the triphenyl tetrazolium chloride (TTC) method as described by Clemensson-Lindell and Persson [56] and Zhang et al. [57], with some modifications. A total of 0.5 g of fresh root tips was weighed and placed in a test tube, and a TTC solution of 0.4% and 5 mL of 0.1 M phosphate buffer at pH 7.5 were added and incubated for 2 h. The reaction was terminated with 1 M of sulfuric acid. The red root system was extracted and ground to 10 mL with acetone, and the absorbance at 485 nm was measured.

### 4.6. AE Activity Assays

A total of 0.3 g of leaf sample was ground in 3 mL of ice-cold 25 mM HEPES buffer (pH 7.8) that contained 0.2 mM EDTA, 2 mM ascorbic acid (AsA), and 2% polyvinylpyrrolidone (PVP) for the AE assays. The homogenates were centrifuged at 12,000× *g* for 20 min at 4 °C, and the supernatants were collected to determine the enzymatic activity. The activity of superoxide dismutase (SOD) was measured in a reaction mixture that contained 0.1 mM EDTA, 2 μM riboflavin, 13 mM methionine, 75 μM nitroblue tetrazolium (NBT), 50 μL enzyme aliquot, and 50 mM of pH 7.8 phosphate buffer [58]. One unit of SOD activity was defined as the amount of enzyme required to cause a 50% inhibition of the rate of reduction in NBT at 560 nm. With some modifications, the method of Cakmak and Marschner [59] was used to determine the guaiacol peroxidase (G-POD) activity. The reaction mixture contained 1.0 mM H_2_O_2_, 0.05% guaiacol, 100 μL enzyme extract, and 25 mM phosphate buffer of pH 7.0. The G-POD activity was determined by measuring the increase at 470 nm caused by the oxidation of guaiacol (E = 26.6 mM cm^−1^). Catalase (CAT) was assayed as described by Durner and Klessig [60], and the decrease in the absorbance at 240 nm for 1 min following the decomposition of H_2_O_2_ was used to determine the activity. Ascorbate peroxidase (APX) was measured as described by Nakano and Asada [61]. The rate of ascorbate oxidation was measured at 290 nm. The data of all the enzymes were expressed as specific activity with the protein content determined using the method of Bradford [62].

### 4.7. Measurements of Proline, Soluble Protein, and MDA Contents

A total of 0.5 g of leaf sample from each group was homogenized in 3% (*w*/*v*) 5-sulfosalicylic acid, and the homogenate was then filtered through filter paper to determine the level of free proline [63]. After the addition of ninhydrin acid and glacial acetic acid, the mixture was heated at 100 °C in a water bath for 1 h. The reaction was terminated in an ice bath, and then the mixture was extracted with toluene. The absorbance of fraction with toluene aspired from the liquid phase was measured at 520 nm, and a calibration curve was used to determine the proline concentration [64].

The supernatant that was used for the analysis of enzyme activity was also collected to assay the soluble proteins. An aliquot of the extract (20 µL) was mixed in 3 mL Coomassie brilliant blue solution, which was used to determine the protein content at 595 nm as described by Bradford [62] using bovine serum albumin as the standard.

The content of MDA was measured as described by Hodges et al. [65]. A total of 0.3 g leaf samples was ground with 3 mL ice-cold 25 mM HEPES buffer at pH 7.8 that contained 0.2 mM EDTA and 2% PVP. The homogenates were centrifuged at 12,000× *g* for 20 min at 4 °C, and the resulting supernatants were mixed with 10% TCA that contained 0.65% 2-thiobarbituric acid (TBA) and heated at 95 °C for 25 min. The content of MDA was measured at 532 and 600 nm by subtracting the absorbance of a solution that contained a plant extract incubated without TBA from an identical solution that contained TBA.

### 4.8. PME Determination

Fresh sweet pepper seedlings were heated to denature the enzymes at 105 °C for 2 h and then dried at 80 °C for 3 days. The PME was determined as described by Song et al. [66], with modifications. The total nitrogen (TN) was analyzed using the near-infrared method. Molybdenum (Mo) was determined using inductively coupled plasma mass spectrometry (ICP-MS). The contents of chloride (Cl), phosphorus (P), potassium (K), calcium (Ca), magnesium (Mg), zinc (Zn), sulfur (S), iron (Fe), manganese (Mn), boron (B), and copper (Cu) were determined by flow analysis using inductively coupled plasma atomic emission spectrometry (ICP-AES).

### 4.9. Statistical Analysis

A one-way analysis of variance (ANOVA) was conducted using SAS v. 9.3 (SAS Institute Inc., Cary, NC, USA). Each value was presented as the mean ± standard deviation (SD) with a minimum of three replicates. Differences between the treatment means were tested using the Least Significant Difference (LSD) method at α = 0.05 level of significance. The figures were plotted using Origin 8.5 (OriginLab, Northampton, MA, USA).

## 5. Conclusions

We demonstrated that sweet pepper grew better in the medium EC treatment of 2.9 dS m^−1^ compared with the other EC treatments. Plants in the EC2.9 treatment tended to have a high photosynthetic rate, more biomass, high root activity, a good response of physiological changes, and a suitable content of PME. Sweet pepper plants can endure EC that is too low or too high for a period of time, but it would clearly inhibit the growth of plants and injure them, and the suppressed growth of very high EC treatment is more serious than one that was very low. It is more effective to irrigate with EC < 5.8 dS m^−1^, since the mineral elements may not be effectively absorbed owing to the low root activity, while the resulting fertilizer waste, salinity stress, high MDA content, restricted growth, and photosynthesis decrease. Plants have to adjust to the stress conditions by increasing the activities of antioxidant enzymes, such as APX and G-POD, and accumulate osmolytes, such as soluble protein and proline. When plants are exposed to low EC for long periods, the inadequacy of PME, such as N and Mg, decreases the synthesis of Chl and Car, which results in low amounts of photosynthesis and plant growth. Plants also need to increase the antioxidant enzymes activities of APX and G-POD to adjust to the nutrient deficiency stress. A suitable EC in nutrient solution is very important for the effective growth of sweet pepper.

## Figures and Tables

**Table 1:** Effects of different EC on the growth of sweet pepper after 25 days of treatment.

Treatment	Plant Height (cm)	Stem Diameter (cm)	Shoot Fresh Weight (g)	Shoot Dry Weight (g)	Shoot Moisture Content (%)
EC0.72	57.0 ± 1.58 b	0.85 ± 0.050 b	94.4 ± 6.89 c	13.43 ± 0.94 b	85.77 ± 0.14 c
EC1.45	60.6 ± 1.95 a	0.89 ± 0.037 a	114.3 ± 7.52 ab	15.14 ± 0.80 ab	86.74 ± 0.18 b
EC2.9	62.4 ± 1.14 a	0.90 ± 0.060 a	125.3 ± 7.54 a	16.09 ± 2.17 a	87.17 ± 0.26 a
EC5.8	56.4 ± 1.14 b	0.84 ± 0.032 b	107.5 ± 1.36 b	15.36 ± 0.05 a	85.71 ± 0.19 c
EC11.6	45.0 ± 2.35 c	0.79 ± 0.019 c	64.0 ± 4.28 d	10.55 ± 0.73 c	83.50 ± 0.22 d
EC17.4	39.2 ± 1.30 d	0.71 ± 0.036 d	50.8 ± 5.49 e	9.17 ± 0.97 c	81.93 ± 0.31 e

Data represent the mean ± SD (*n* = 3). EC0.72, EC1.45, EC2.9, EC5.8, EC11.6, and EC17.4 represent the different nutrient solution electrical conductivities of 0.72 dS m^−1^, 1.45 dS m^−1^, 2.9 dS m^−1^, 5.8 dS m^−1^, 11.6 dS m^−1^, 17.4 dS m^−1^. Different letters indicate significant differences at α = 0.05 based on the Least Significant Difference test.

**Table 2:** Effects of different EC on soluble protein, malondialdehyde (MDA), and proline contents of sweet pepper after 25 days of treatment.

Treatment	Soluble Protein Content (mg g^−1^ FW)	MDA Content (mg g^−1^ FW)	Proline Content (μg g^−1^ FW)
EC0.72	2.03 ± 0.14 e	1.35 ± 0.11 d	3.70 ± 0.19 d
EC1.45	3.11 ± 0.07 d	1.58 ± 0.03 c	5.11 ± 0.67 d
EC2.9	3.44 ± 0.08 c	1.24 ± 0.07 d	9.83 ± 0.53 d
EC5.8	3.62 ± 0.05 b	1.61 ± 0.07 c	23.87 ± 2.18 c
EC11.6	3.97 ± 0.10 a	1.85 ± 0.11 b	111.05 ± 1.57 b
EC17.4	3.68 ± 0.07 b	2.06 ± 0.07 a	153.40 ± 7.82 a

EC0.72, EC1.45, EC2.9, EC5.8, EC11.6, and EC17.4 instead of the different nutrient solution electrical conductivities of 0.72 dS m^−1^, 1.45 dS m^−1^, 2.9 dS m^−1^, 5.8 dS m^−1^, 11.6 dS m^−1^, and 17.4 dS m^−1^. The data represent the mean ± SD (*n* = 3). Different letters indicate significant differences at α = 0.05 based on the Least Significant Difference test.

**Table 3:** Element components in the mother NS in different tanks (A, B).

A	kg/1000 L	A	g/1000 L	B	kg/1000 L
Ca(NO_3_)_2_·4H_2_O	130	MnSO_4_·H_2_O	175	KNO_3_	50
EDPA-Fe (13%Fe)	1.4	ZnSO_4_·7H_2_O	125	KH_2_PO_4_	25
		Na_2_B_4_O_7_·4H_2_O	375	K_2_SO_4_	15
		CuSO_4_·5H_2_O	25	MgSO_4_·7H_2_O	50
		Na_2_MoO_4_·2H_2_O	15		

## Data Availability

All data used during the study are available from the author Xiaotao Ding by request (e-mail: xiaotao198108@163.com).

## Appendix A

**Table A1:** Repeated measure ANOVA of the EC treatments, days of measurement, and their interactions on the net photosynthetic rate (*P*_n_), stomatal conductance (*G*_s_), intercellular CO_2_ concentration (*C*_i_), and transpiration rate (*T*_r_).

Source of Variation	*P*_n_		*G*_s_		*C*_i_		*T*_r_	
	*F* Value	*p* Value	*F* Value	*p* Value	*F* Value	*p* Value	*F* Value	*p* Value
EC treatment	148.84	<0.001	141.59	<0.001	128.90	<0.001	119.93	<0.001
Date	15.34	<0.001	30.15	<0.001	34.63	<0.001	16.45	<0.001
EC treatment× Date	5.75	<0.001	4.26	<0.001	2.54	<0.001	3.05	<0.001
